# Phenotype-genotype analysis of CYP2C19 in Colombian mestizo individuals

**DOI:** 10.1186/1472-6904-7-6

**Published:** 2007-07-11

**Authors:** Carlos Isaza, Julieta Henao, José H Isaza Martínez, Juan C Sepúlveda Arias, Leonardo Beltrán

**Affiliations:** 1 Grupo de Investigación en Farmacogenética, Universidad Tecnológica de Pereira, Facultad de Ciencias de la Salud, La Julita, Pereira, Colombia, South America; 2 Grupo Polifenoles UTP-CENIVAM, Facultad de Tecnología, Escuela de Química, Universidad Tecnológica de Pereira, Pereira, Colombia, South America

## Abstract

**Background:**

Omeprazole is metabolized by the hepatic cytochrome P450 (CYP) 2C19 enzyme to 5-hydroxyomeprazole. CYP2C19 exhibits genetic polymorphisms responsible for the presence of poor metabolizers (PMs), intermediate metabolizers (IMs) and extensive metabolizers (EMs). The defective mutations of the enzyme and their frequencies change between different ethnic groups; however, the polymorphism of the *CYP2C19 *gene has not been studied in Colombian mestizos. The aim of this study was to evaluate the genotype and phenotype status of CYP2C19 in Colombian mestizos, in order to contribute to the use of appropriate strategies of drug therapy for this population.

**Methods:**

189 subjects were genotyped using the multiplex SNaPshot technique and a subgroup of 44 individuals received 20 mg of omeprazole followed by blood collection at 3 hours to determine the omeprazole hydroxylation index by HPLC.

**Results:**

83.6%, 15.3% and 1.1% of the subjects were genotyped as EMs, IMs and PMs, respectively. The frequencies of the CYP2C29*1 and CYP2C19*2 alleles were 91.3% and 8.7% respectively whereas the *3, *4, *5, *6 and *8 alleles were not found. No discrepancies were found between the genotype and phenotype of CYP2C19.

**Conclusion:**

The frequency of poor metabolizers (1.1%) in the Colombian mestizos included in this study is similar to that in Bolivian mestizos (1%) but lower than in Mexican-Americans (3.2%), West Mexicans (6%), Caucasians (5%) and African Americans (5.4%). The results of this study will be useful for drug dosage recommendations in Colombian mestizos.

## Background

The CYP2C19 isoenzyme, a member of the superfamily of xenobiotic enzymes of cytochrome P-450, is responsible for the metabolism of several therapeutically important drugs, such as proton-pump inhibitors (omeprazole, lansoprazole, pantoprazole), antidepressants (citalopram, imipramine), benzodiazepines (diazepam, flunitrazepam), propranolol and proguanil [1-3].

The *CYP2C19 *gene polymorphism divides populations in three phenotypic subgroups: extensive metabolizers (EMs), intermediate metabolizers (IMs), and poor metabolizers (PMs). The enzymatic deficiency is inherited as an autosomal recessive trait [4].

The frequencies and types of alleles vary between ethnic groups. Thus, 13 to 23% of Orientals are PMs and the CYP2C19*2 and *3 alleles account for 99% of them in this ethnic group [5], whereas in Caucasians, with a percentage of PMs near to 5% of the population, only the *2 allele is common although other variants have been described [6-9]. In Black population some new mutations (*9, *10, *12) have been reported and the frequency of PMs individuals is 5.4% [10,11]. The clinical consequences of the *CYP2C19 *gene polymorphisms have not been fully understand but can be illustrated with the case of proton-pump inhibitors. The EMs metabolizes these drugs at a speed that requires doses up to four times greater than PMs to reach similar serum concentrations and effects [12,13].

Several studies carried out in different ethnic groups have shown that the efficacy of proton-pump inhibitors is related to the *CYP2C19 *genotype, explaining the differences in terms of therapeutic efficacy observed between EMs and PMs individuals [14-19]. Nevertheless, the resistance of *H. pylori *to antimicrobial and others non-genetic factors such as age, liver disease and enzymatic inhibition or induction by drugs are also important causes of therapeutic failure [3,20,21].

Although the genotype-phenotype correlation for CYP2C19 using omeprazole as "probe drug" has been widely studied, there are no data regarding South American Mestizos [22,23]. Therefore, the aim of this study was to evaluate the genotype and phenotype status of CYP2C19 in Colombian mestizos, in order to contribute to the use of appropriate strategies of drug therapy for this population. Colombian population is divided ethnically into four main groups: the Mestizo (56%), White (30.1%), Black (10.5%) and Amerindian (3.4%) people. The Colombian mestizo population is an admixture between Amerindian, Hispanic and African descent [24].

## Methods

### Subjects

Healthy unrelated Colombian mestizos (n = 189) participated in the study. None of the volunteers included in the phenotyping test (n = 44) had a history of allergy to omeprazole or other drugs, alcohol abuse, drug addiction or a smoking habit of more than 15 cigarettes/day. The participants were not allowed to take any medication during the previous week before and during the study [25]. The experimental protocol was approved by the Ethics Committee of Universidad Tecnológica de Pereira, Colombia. Written informed consent was obtained from all participating subjects.

### *CYP2C19 *genotyping

A multiplex primer-extension assay that simultaneously detects the six CYP2C19 alleles using the ABI Prism^® ^SNaPshot™ ddNTP Primer-Extension Assay was used [26]. Buccal swabs were obtained from experimental subjects and the material was deposited onto FTA blood cards, dried at room temperature and stored for DNA extraction as reported [27]. The DNA was used to amplify five fragments of the *CYP2C19 *gene corresponding to exons 1 (410 bp), 2–3 (719 bp), 4 (310 bp), 5 (410 bp) and 9 (529 bp); which include the six Single Nucleotide Polymorphisms (SNPs) studied. Amplification was carried out on a DNA thermal cycler PBX2 (Thermo Electron Corporation), according to previously described techniques [28]. PCR products were electrophoresed in 2% agarose gels and the bands purified using the GFX purification kit (Amersham Pharmacia Biotech). The amplification products were used as templates for the multiplex reactions in order to detect the wild-type allele CYP2C19*1 and the *2, *3, *4, *5, *6 and *8 mutations.

The minisequencing was based on the method proposed by Bender *et al *[26], with detection primers present in the reaction at a concentration of 1.5 pmoles. The amplification products were injected onto the DNA ABI prism 3100 Avant Genetic Analyzer ant the results were analyzed using the GeneMapper Analysis Software (PE Applied Biosystems).

### CYP2C19 phenotyping

The omeprazole hydroxylation index (HI) was determined in 44 subjects previously genotyped. After an overnight fast, each subject took 20 mg omeprazole (Tecnoquímicas, Cali, Colombia) orally. Then, 3 h after ingestion of the drug, 5 ml of venous blood was collected from an antecubital vein into tubes with anticoagulant. Plasma was separated after centrifugation and stored frozen at -20°C until analysis. The concentration of omeprazole and 5-hydroxy omeprazole in plasma was measured by HPLC as described by Gonzalez *et al *[29], with some modifications. Omeprazole and 5-hydroxy omeprazole were purchased from Sigma-Aldrich Co (St. Louis, USA) and SynFine Research Inc. (Ontario, Canada), respectively. To avoid recovery problems, standards were prepared by spiking blank plasma with omeprazole and 5-hydroxy omeprazole to get concentrations of 0.1, 0.25, 1.0, 3.0 and 5.0 μg/mL and processed by solid phase extraction (SPE) in the same way as samples. Standards and samples (2 mL) were submitted to SPE using 500 mg RP-18 cartridges (Merck) previously activated with AcCN (3 mL), followed by three portions of 3 mL of phosphate buffer (0.05 M, pH 7.2). After sample application the cartridge was washed sequentially by three 0.5 mL portions of phosphate buffer and a 0.5 mL portion of phosphate buffer-AcCN (80:20), drying after each washing. Analytes were eluted with phosphate buffer-AcCN (10:90). HPLC analysis was carried out in a Jasco HPLC 2000 plus Series System equipped with a PU-2089 Plus Quaternary Gradient Pump, an AS-2059 Plus Intelligent Autosampler, a CO-2065 Plus Column Oven, a MD-2015 Plus Intelligent Diode Array Detector, and a LC Net II/ADC, controlled by EZChrom Elite Software version 3.16. Each standard and sample were injected (25 μL) into an Ultra Aqueous RP-18 Restek analytical column (3 μm particle size, 100 × 3.2 mm I.D.), eluting in isocratic mode with phosphate buffer-AcCN (70:30) as mobile phase at 0.5 mL/min flow rate. Calibration curve and quantification were performed at 298 nm by external standard, with linear fit showing a coefficient of determination (*r*^2^) of 0.9993 for omeprazole and 0.9992 for 5-hydroxyomeprazole in the range from 0.1 to 5 μg/mL. Omeprazole and its metabolite 5-hydroxyomeprazole eluted at 9.80 min and 3.67 min, respectively. Detection limits were 0.026 μg/mL for omeprazole and 0.01 μg/mL for 5-hydroxyomeprazole. Intraday coefficients of variation for omeprazole and 5-hydroxyomeprazole were 5% and 4%, respectively. Individuals with a metabolic ratio (MR) ≥ 0.63 were phenotyped as PMs.

### Statistical analysis

Allelic frequencies were calculated according to the number of alleles observed and the number of chromosomes examined. The Hardy-Weinberg equilibrium was established using the Chi-square test. The MR was calculated as log_10 _(omeprazole/hydroxyomeprazole) [21,29,30]. The normality of the MR distribution was evaluated by the D'Agostino test. A probit plot was used to confirm the bimodal distribution of the MR. 95% confidence intervals were used and the level of statistical significance was set at *p *< 0.05. Statistical analysis was conducted using SPSS 10.0 for Windows (SPSS, USA) and the GraphPad Prism 5.0 software (GraphPad Software Inc. San Diego, Ca, USA).

## Results

Allelic and genotypic frequencies for *CYP2C19 *were determined in 189 Colombian mestizos (51% men; age rank 18–56 years), data are shown in table 1. The frequency of the native allele CYP2C19*1 was the highest (91.3%) followed for the CYP2C19*2 allele (8.7%). The non functional alleles *3, *4, *5, *6 and *8 were not detected. Among the genotyped groups, 83.6% of the subjects were homozygous for CYP2C19*1, 15.3% were heterozygous for CYP2C19*1 (*1/*2) with one functional allele, and 1.1% of the subjects were homozygous for the non functional allele CYP2C19*2 (*2/*2). Thus, the frequencies of EMs, IMs and PMs were 83.6%, 15.3% and 1.1% respectively. The distribution of genotypes for *CYP2C19 *were in Hardy-Weinberg equilibrium (Chi = 0.26; df = 2; P = 0.88).

**Table 1:** Allelic and genotypic frequencies of *CYP2C19 *in Colombian Mestizo individuals (n = 189). CI: confidence interval.

***CYP2C19 *Alleles**	**Number of alleles**	**Frequency (%) (95% CI)**
*1	345	91.3 (88.5–94.1)
*2	33	8.7 (5.9–11.5)
*3	0	0
*4	0	0
*5	0	0
*6	0	0
*8	0	0

***CYP2C19 *genotypes**	**Number of subjects**	**Frequency (%) (95% CI)**

*1/*1	158	83.6 (78.3–88.9)
*1/*2	29	15.3 (10.2–20.4)
*2/*2	2	1.1 (0.4–3)

**Figure 1:**
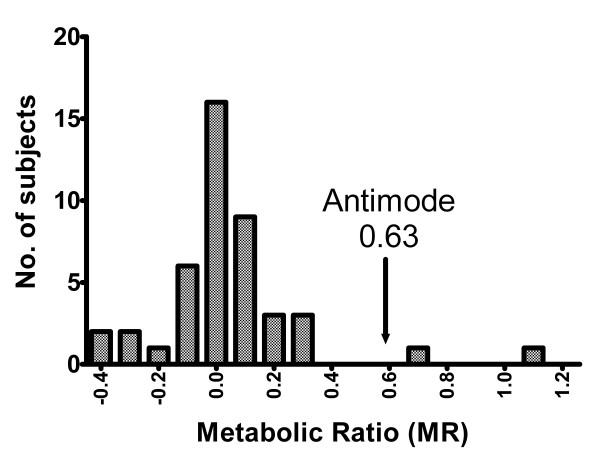
Frequency distribution of the hydroxylation indexes of Omeprazole in Colombian mestizos. The arrow shows the antimode (0.63). Individuals with an MR ≥ 0.63 were phenotyped as PMs.

**Figure 2:**
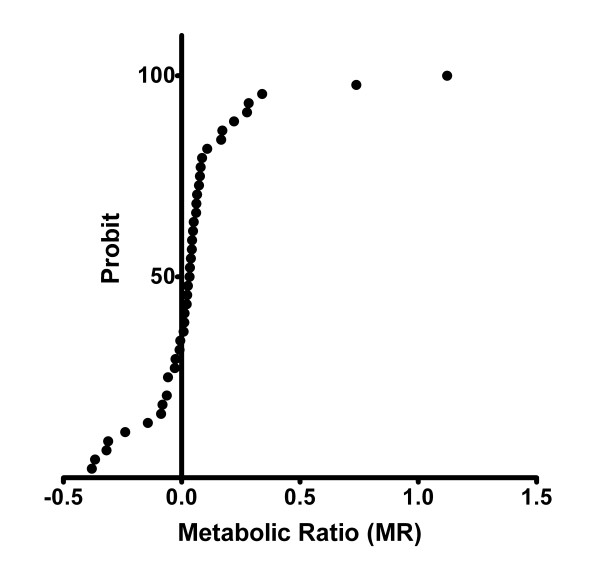
Probit plot obtained by curve fitting of the log MR data from 44 healthy volunteers. The analysis revealed bimodality with the intersecting point at X = 0.63.

The averages of MR [log (omeprazole/hydroxyomeprazole)] for the three genotypes are statistically different (table 2). The figure 1 shows the frequency distribution histogram of the MR for the 44 phenotyped individuals. The D'Agostino test indicated that the data deviated from a normal distribution. The Probit analysis confirmed a bimodal distribution with an antimode of 0.63 (figure 2). For the individuals previously genotyped as EMs/IMs (n = 42), the values of log (omeprazole/hydroxyomeprazole) were in the rank from -0.38 to 0.34, whereas those genotyped as PM (n = 2) were in the rank from 0.738 to 1.24, showing a complete genotype-phenotype concordance between the EMs/IMs and PMs.

**Table 2:** Correlation between CYP2C19 genotype and MR in Colombian Mestizo individuals (n = 44). MR: Metabolic ratio, n: number of subjects, SD standard deviation.

**GENOTYPE**	***n***	**MR (mean ± SD)**	***P***
*1/*1	39	-0.01 ± 0.15	
*1/*2	3	0.23 ± 0.15	
*2/*2	2	0.99 ± 0.35	<0.01

## Discussion and conclusion

Genetic polymorphisms are the more influential factors for individual and interethnic variations in drug responses [31]. The metabolic activity of CYP2C19 is genetically controlled and individuals can be characterized as EMs, IMs or PMs.

This is the first study evaluating the genotype and phenotype status of CYP2C19 in Colombian mestizos. The *CYP2C19 *genetic profile found in this population, with the *2 allele responsible for most of the deficient metabolizers, and the absence of *3, *4, *5, *6 and *8 variants, is similar to Mexican-Americans profile [32], however, the PMs frequency is lower (1,1% *vs. *3,2%). Also, the frequency of PMs found in our study is lower that the ones reported in Caucasians (5%) [33], Blacks (5.4%) [10] and West Mexicans [22], which are genetically related to American mestizos [5,6]. Since Amerindians are also part of the American mestizo ancestor, it is interesting to mention that PMs were not found in Cuna Amerindians of Panama [34], whereas in Bolivian mestizos (an admixture between white and Amerindian populations), the prevalence of PMs is 1% [35].

The pharmacogenetic characterization becomes one of the therapeutic options more cost-effective [36], because it is helpful for both to prevent adverse drug reactions as well as to enhance therapeutic efficiency in the case of drugs with narrow therapeutic index. The genotype-phenotype correlation found in this study, allows us to state that genotyping only the *2 allele would have a high predictive value of the CYP2C19 phenotype between Colombian mestizos. Since pharmacogenetic allows the introduction of personalized pharmacotherapy, according to individual genetic data, the results of this study will be useful for drug dosage recommendations in Colombian mestizos.

For *CYP2C19 *genotyping we used the minisequencing Multiplex SnaPshot technique [26], which could be a safer and more cost-effective strategy than the conventional genotyping techniques based on allele-specific PCR or RFLP (restriction fragment length polymorphism).

## Competing interests

The author(s) declare that they have no competing interests.

## Authors' contributions

CI conceived the study and participated in its design and coordination and wrote the first draft of the manuscript. He also recruited subjects, obtained informed consent, supervised drug administration and carried out the entire statistical analysis. JH and LB collected blood samples and were responsible for the genotyping. LB, JHIM and JCSA were responsible for the HPLC analysis. JCSA also assisted to draft the manuscript. All authors read and approved the final manuscript.

## Pre-publication history

The pre-publication history for this paper can be accessed here:


